# TLR4 induced TRPM2 mediated neuropathic pain

**DOI:** 10.3389/fphar.2024.1472771

**Published:** 2024-09-12

**Authors:** Venkata Kiran Kumar Mandlem, Ana Rivera, Zaina Khan, Sohel H. Quazi, Farah Deba

**Affiliations:** ^1^ Departmental of Pharmaceutical Sciences and Health Outcomes, The Ben and Maytee Fisch College of Pharmacy, University of Texas at Tyler, Tyler, TX, United States; ^2^ Departmental of Neuroscience, University of Texas at Dallas, Richardson, TX, United States; ^3^ Department of Biology, Division of Natural and Computation Sciences, Texas College, Tyler, TX, United States

**Keywords:** inflammation, neuropathic pain, lipopolysaccharide reactive oxygen species (ROS), nicotinamide adenine dinucleotide phosphate (NADPH) oxidase (NOX), TRPM2 and TLR4

## Abstract

Ion channels play an important role in mediating pain through signal transduction, regulation, and control of responses, particularly in neuropathic pain. Transient receptor potential channel superfamily plays an important role in cation permeability and cellular signaling. Transient receptor potential channel Melastatin 2 (TRPM2) subfamily regulates Ca^2+^ concentration in response to various chemicals and signals from the surrounding environment. TRPM2 has a role in several physiological functions such as cellular osmosis, temperature sensing, cellular proliferation, as well as the manifestation of many disease processes such as pain process, cancer, apoptosis, endothelial dysfunction, angiogenesis, renal and lung fibrosis, and cerebral ischemic stroke. Toll-like Receptor 4 (TLR4) is a critical initiator of the immune response to inflammatory stimuli, particularly those triggered by Lipopolysaccharide (LPS). It activates downstream pathways leading to the production of oxidative molecules and inflammatory cytokines, which are modulated by basal and store-operated calcium ion signaling. The cytokine production and release cause an imbalance of antioxidant enzymes and redox potential in the Endoplasmic Reticulum and mitochondria due to oxidative stress, which results from TLR-4 activation and consequently induces the production of inflammatory cytokines in neuronal cells, exacerbating the pain process. Very few studies have reported the role of TRPM2 and its association with Toll-like receptors in the context of neuropathic pain. However, the molecular mechanism underlying the interaction between TRPM2 and TLR-4 and the quantum of impact in acute and chronic neuropathic pain remains unclear. Understanding the link between TLR-4 and TRPM2 will provide more insights into pain regulation mechanisms for the development of new therapeutic molecules to address neuropathic pain.

## 1 Introduction

Pain is defined as a characteristic phenomenon caused by noxious stimuli from pathogenic microorganisms such as bacteria, viruses, and fungi and from injury caused by various forms of mechanical and thermal stimuli. The physiological response to these conditions results in pain. Relief from pain is crucial for an organism’s survival and protection because pain serves as a warning signal for potential harm or injury. It triggers reflexes and behaviors that help avoid or minimize damage, promoting healing and preventing further injury. Without effective pain relief mechanisms, an organism could suffer prolonged stress, which can lead to immune suppression, impaired function, and reduced chances of survival. Therefore, managing pain is not only important for comfort but also vital for the overall wellbeing and preservation of life. Normal pain symptoms involve allodynia, which is non-painful stimuli, such as a light touch or mild temperature change, whereas hyperalgesia refers to an increased sensitivity to painful stimuli generated during minor injury. The symptoms of hypersensitivity involve both allodynia and hyperalgesia, which are intense responses to sensory stimuli, resulting in enhanced sensitivity and discomfort. Persistent and extreme sensitivity are also referred to as chronic pain (Loeser and Melzack, 1999; Świeboda et al., 2013). Acute pain condition is a protective mechanism in which tissue stimulates the healing process. Beyond any alteration in the pain pathway and duration leads to hypersensitivity and intolerable or chronic pain.

Depending on the duration of symptoms, pain is classified as acute pain (<3 months) and chronic pain (>3 months). Pain is also classified into many subtypes such as anatomical pain, physiological pain, pathological pain, deep pain, vascular pain, bone and joint pain, myalgia, organ pain, wired pain, neuralgia, causalgia, convolutional pain, and phantom pain, depending upon the stimuli, location, physiological and social factors (Świeboda et al., 2013; Sela et al., 2002).

According to the International Association for the Study of Pain defines, neuropathic pain is a pain caused by a lesion or disease affecting the somatosensory nervous system. As per the International classification of diseases, conditions for peripheral neuropathic pain include trigeminal neuralgia, polyneuropathy, peripheral nerve injury, and painful radiculopathy. Conditions for central neuropathy include pain caused by brain or spinal cord injury, post-stroke pain, and pain associated with multiple sclerosis (Scholz et al., 2019). Neuropathic pain is associated with damage to the nerves, including central, peripheral, and somatosensory nervous systems. It involves both positive and negative symptoms. Positive symptoms include paresthesia, dysesthesia, superficial pain, allodynia, and hyperalgesia. Negative symptoms include tactile hypoesthesia, thermal hypoesthesia, and pinprick hypoalgesia (Baron, 2006). Neuropathic pain is considered chronic pain that affects the quality of normal life of an individual. Trigeminal neuralgia, diabetic neuropathic pain, cancer pain, and sciatica are some examples of chronic pain. Many of the neuronal complex structures are involved in the activation, transmission, regulation, and healing processes (Backonja, 2003). Nerve endings, dorsal root ganglia, microglia, Schwann cells, and thalamus are the important structures that are involved in chronic pain. Structural changes, numerous cellular interactions, and signaling processes contribute to the nociceptive pathway, including ion channels, receptors, immune cells, and microglia (Finnerup et al., 2020; Kerstman et al., 2013). The transition from acute neuropathic pain to chronic pain is a complex process and involves multiple signaling pathways, central and peripheral processes, and inherited and environmental risks.

Neuroinflammation is an immune-mediated inflammatory response elicited by various stimuli. Cytokines released in response to the inflammation regulate oxidative stress and mediate the injury and/or repair process (Myers et al., 2006). Sensitization of nerve endings and other inflammatory molecules mediate the pain process in which ion channels play an important role in mediating pain, inflammation, and repair depending on Ca^2+^ ion influx (Kuffler, 2020). Many of the ion channels are regulated either by a change in the voltage (cation/anion exchange) or receptor-mediated responses. These ion channels respond to changes in the cellular external and internal environments, thereby eliciting a physiological response (Bouali-Benazzouz et al., 2021; Vicario et al., 2020). Ion channels exhibit regulatory properties of immune cells, metabolism, and inflammation pathways (Ruck et al., 2022; Bittner et al., 2014). Apart from the homeostatic functions, ion channels may also be involved in the pathological mechanisms of neuronal inflammation that leads to pain. Membrane ion channels that govern sodium, potassium, and calcium ions and their modulation by various inflammatory mediators are involved in neuropathic pain.

Inflammatory responses and oxidative stress are regulated by molecular inhibitors and activators, Toll-like-receptor (TLR-4) is a transmembrane pattern recognition receptor (Fehder et al., 2007), Nicotinamide Adenine Dinucleotide phosphate (NADPH) oxidase (NOX), and Transient receptor potential (TRP), an ion channel and a well-known receptor for immune responses. TLR-4 is associated with many pathological conditions with chronic neuropathic pain. TRPM2 is a calcium-permeable cation channel in sensory neurons. NOX is part of a family of enzymes that includes NOX1, NOX2, NOX3, NOX4, NOX5, DUOX1, and DUOX2. NOX enzymes are known for immune defenses in which Reactive Oxygen Species (ROS) production by phagocytic cells to kill pathogens (Vermot et al., 2021). TRPM2 channels are activated by ROS produced by NOX, which leads to calcium influx. This calcium signaling boosts NOX activity, which amplifies ROS production and oxidative stress. This pathway is associated with several diseases, including neurodegenerative diseases and chronic pain (Görlach et al., 2015).

**FIGURE 1:**
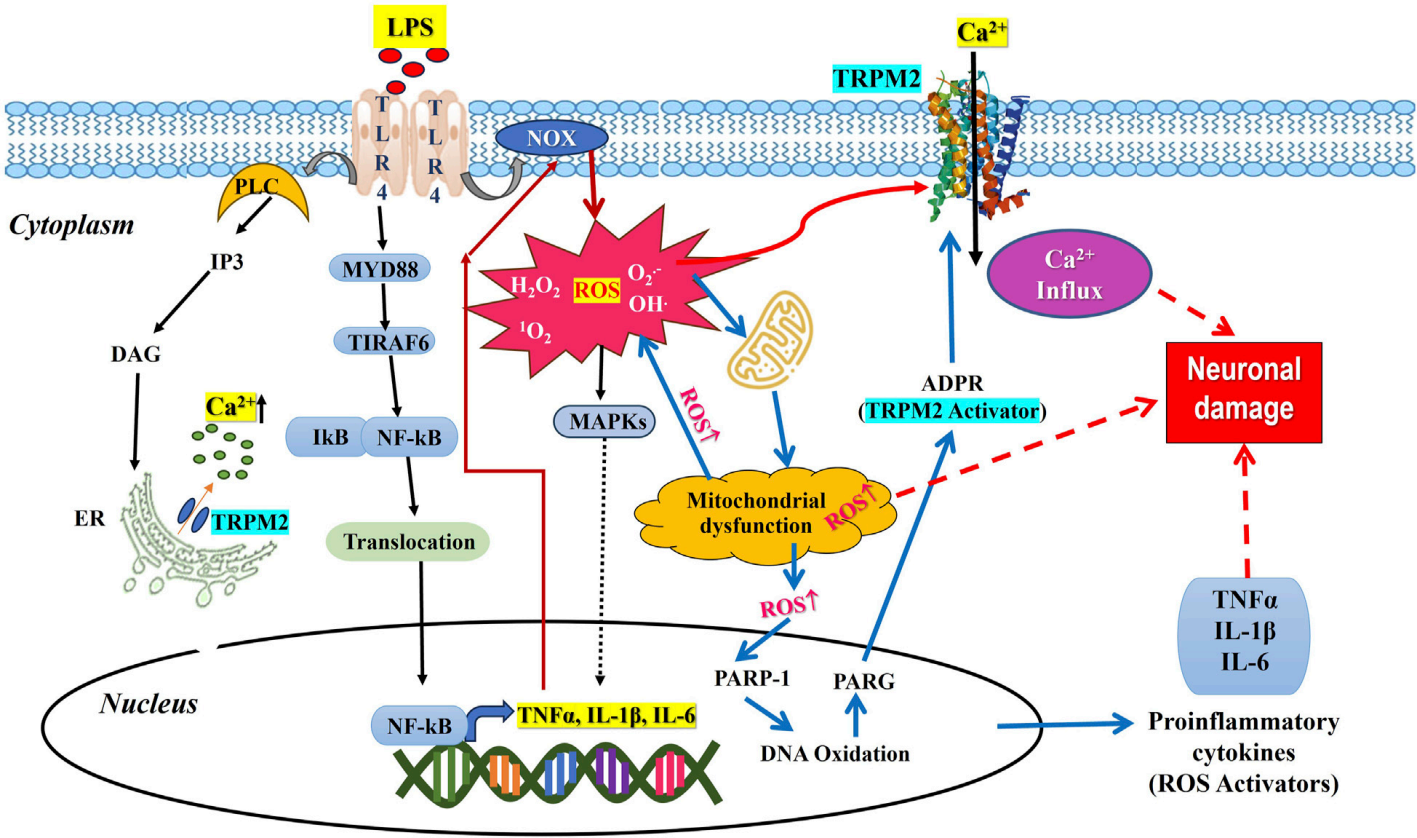
Mechanism of TLR4 induced TRPM2 mediated neuropathic pain.

The present review aims to address pathways in which oxidative stress and inflammatory reactions by TLR-4, NOX, and TRPM2 implicate pain (Figure 1). These findings highlight the biological importance of TRPM2 and TLR-4 for elucidating pain pathways due to producing ROS and inflammatory cytokines, which are important in regulating oxidative stress and inflammation in cells. TRPM2 inhibitors could decrease oxidative stress-induced injuries and neuropathic pain in a wide variety of ways. TLR-4 antagonists may decrease inflammatory responses and alleviate chronic inflammation. The discovery of the mechanisms by which TRPM2 activation drives nerve damage and inflammation, and its association with TLR-4 can show new pathways; such understanding may become apparent as an innovative therapy for disorders assumed to cause or intensify neuropathic pain.

## 2 Ion channels and their role in diseases

The process of recognizing pain involves the recognition of pain signals by sensory neurons, initiation of nerve impulses, and transforming the noxious stimuli to pain response. These nociceptive neurons are specialized in the recognition of noxious stimuli, of which ion channels on cell membrane play an important role in intermembrane transporters of various ions, thereby regulating various physiological functions ranging from excitation of tissue transportation, cell migration tumorigenesis, atherosclerosis, and apoptosis (Kew and Davies, 2010; Schwab et al., 2012; Jentsch, 2016). Voltage-gated ion channels are widely expressed in all tissues, including neurons, such as sodium Nav1.8 and Nav1.9 channels (Giniatullin, 2020). Other ion channels such as purinergic receptor, TRPV1(vanilloid)/A1 (ankyrin), TRPC (canonical), TRPM2, and voltage-gated (sodium, calcium, and potassium) channels are also involved in nociception. Many of the neuronal diseases, including neuropathic pain, are linked to ion channel dysfunction and neuroinflammation (Eren-Koçak and Dalkara, 2021).

## 3 TRP channel

Transient receptor potential (TRP) channels are widely distributed throughout the tissues. In 1969, TRP was discovered in *drosophila* and subsequently in humans (Montell and Rubin, 1989; Samanta et al., 2018). The TRP channels superfamily were extended up to 48 members and their major role was found in cation permeability and cellular sensory signaling. These channel proteins span the transmembrane with a C-terminal intracellular domain and N-terminal intracellular domain (Kassmann et al., 2013), which are non-selective ion channels that regulate Ca^2+^, Mg^2+^ influx, and other monovalent cations. These TRP channels can be activated in cellular organelles, such as endoplasmic reticulum, lysosomes, Golgi apparatus, and synaptic vesicles by Ca^2+^ influx. TRP channels are also involved in the regulation of osmotic pressure, temperature, pH oxidation-reduction vascular tone, proliferation, mechanosensing, proliferation, apoptosis, and angiogenesis (Gees et al., 2010). Therefore, TRP channels respond to various stimuli such as stress, mediators, hormones, chemicals, and environmental factors (Lolignier et al., 2016; Kaneko and Szallasi, 2014). Changes in the channel function result in many disorders, such as retinal, skin, cardiac, neuronal, and vascular, forming the basis of mutation in the genes responsible for coding for TRP channel (Tsagareli and Nozadze, 2020). TRP Channels have been implicated in various neurodegenerative diseases such as Alzheimer’s, Parkinson’s, and Huntington’s diseases (Rather et al., 2023). TRP channels are proven to play as central elements of nociception, thereby acting as potential targets for alleviating neuropathic pain. Modulating the TRP channel function offers a wide scope for altering cellular functions, thereby increasing the research interest. Modulation of TRP channels has emerged as a novel field of therapeutic strategy for the treatment of pain, especially neuropathic pain. Many small molecule compounds that modulate TRP channels have entered clinical trials aiming to treat a variety of neuronal diseases (Table 1).

**TABLE 1:** Present and ongoing TRP ion channels clinical drugs used for pain pathology.

Agent	Antagonists/Activators	Indications	Status	Notes	Ref.
Gabapentin	TRPV1 antagonist (Indirect)	Neuropathic pain, epilepsy	Approved	Gabapentin combined with Capsaicin enters the neurons through TRPV1 to access Voltage-gated channels	Biggs et al. (2015)
Pregabalin	TRPV1 antagonist (Indirect)	Neuropathic pain, fibromyalgia, epilepsy	Approved	Pregabalin is used for its antagonistic activity at Voltage-gated Ca^2+^ channels and binds to alpha-2-delta	Onakpoya et al. (2019)
Capsaicin	TRPV1 activator	Neuropathic pain	Approved	Capsaicin desensitizes, calcium-permeable TRPV1 channels and relieves pain	Pasierski and Szulczyk (2022)
SB-705498	TRPV1 antagonist	Rhinitis Pain relief	Clinical Phase II	SB-705498 relieved pain by suppressing response to skin stimulation by TRPV1 receptor	Lambert et al. (2009)
Resiniferatoxin	TRPV1 activator	osteoarthritic pain	Clinical Phase II	Resiniferatoxin through injection desensitizes TRPV1	Szallasi (2023)
Tramadol	TRPV1 antagonist (Indirect)	neuropathic pain	Approved	Tramadol relieves Neuropathic Pain by regulating mediators in pain signaling (TRPV1)	Quan et al. (2022)
XEN-D0501	TRPV1 receptor antagonist	Chronic pain	Clinical Phase II	XEN-D0501 is used as a temperature regulator by blocking the activation of TRPV1 receptor	Round et al. (2011)
Topiramate	TRPV1 antagonist (Indirect)	Migraine	Approved	Topiramate is used as anti-migraine, activates Na^+^ channels and Ca^2+^ channels	Fan et al. (2018)
Zonisamide	TRPV1 antagonist (Indirect)	Migraine	Clinical Phase II	Zonisamide is used a anti-migraine, activates Na^+^ channels and Ca^2+^ channel	Asiri and Hassan (2023)
GRC 6211	TRPV1 antagonists	Osteoarthritis pain, chronic pain	Clinical Phase II	GRC 6211 suppresses neurogenic detrusor overactivity	Santos-Silva et al. (2012)
PF-05105679	TRPM8 antagonists	Cold Pain Sensitivity	Clinical Phase II	PF-05105679 is used to regulate body temperature	Gosset et al. (2017)
AMG-333	TRPM8 antagonists	Migraine	Clinical Phase II	TRPM8 is activated. By cold and AMG-333 is a highly selective antagonist	Horne et al. (2018)
KRP-2529	TRPM8 antagonists	hypersensitive bladder disorders	Preclinical	TRPM8 antagonist reduces hypersensitivity by blocking TRPM8 activity	Aizawa et al. (2018)
RQ-00434739	TRPM8 antagonists	hypersensitive bladder disorders	Preclinical	TRPM8 antagonist reduces hypersensitivity by blocking TRPM8 activity	Aizawa and Fujita (2022)
HC-030031	TRPA1 antagonists	Chronic Pain	Preclinical	HC-030031 reduces pain and inflammation by blocking TRPA1 channels	de Almeida et al. (2021)
GRC-17536	TRPA1 antagonists	Hypersensitivity	Clinical Phase II	TRPA1 antagonists inhibit the activation of the channel that detects pain	Klionsky et al. (2007)
AMG-9090	TRPA1 antagonists	Chronic Pain	Preclinical	TRPA1 antagonist reduces hypersensitivity by blocking TRPA1 activity	Klionsky et al. (2007)
LY3526318	TRPA1 antagonists	Neuropathic Pain	Clinical Phase II	LY3526318 alleviates pain by inhibiting TRPA1 channel activity	Bamps et al. (2023)

## 4 TRPM channel structure and functions

TRPM channels are ubiquitous in nature and widely expressed and distributed in tissues and contribute to health and disease. The three domains of these ion channels are N, C, and channel domain, where the N-terminal is associated with four similar melastatin subunits and a pre-S1 domain to play a role in sensing and channel assembly. The channel domain S4 is specific to the TRPM family and participates in a voltage-sensing-like domain, whereas S5-S6 forms the P-loop, which mainly acts as an ion-conducting pore. The C-Domain is composed of a TRP box, which is highly conserved and maintains channel stability, whereas the coiled-coil domain contains motifs that modulate pore gating. The differences in the homology sequence of the C-terminus divide the TRPM subfamily into four groups: TRPM3/TRPM6/TRPM7, highly permeable to Ca^2+^, TRPM2/TRPM8 nonselective for monovalent and divalent cations, whereas TRPM4/TRPM5 is permeable to monovalent cations. TRPM2/TRPM6/TRPM7 present enzymatic properties at the C terminus, and TRPM2 has an additional domain for nucleoside diphosphate pyrophosphatase, which shares a similar homology with Nudix hydrolase NUDT (-H domain), which exhibit a wide range of functions such as metabolism of nicotinamide adenine dinucleotide (NAD), ADP-ribose, and their derivatives and is characterized by different substrate specificity and intracellular localization. (Samanta et al., 2018; McLennan, 2006). TRPM8 is temperature sensitive and activated by innocuous cold to noxious cold. It is also activated by menthol and icilin, synthetic super-agonist. Both TRPM8 agonists and antagonists, e.g., AMTB, proved to be beneficial in pain pathways (Fernández-Peña and Viana, 2013). TRPM3 is a polymodal nociceptor thought to be involved in the detection of noxious heat (Vriens et al., 2011). TRPM3, together with TRPV1 and TRPA1, mediate acute noxious heat detection in mice (Vandewauw et al., 2018). Given the importance of TRPM channels, there is an increase in research interest in TRPM channels and their role in triggering various diseases, including pain process, cellular osmosis, temperature sensing, cellular proliferation, cancer, apoptosis, endothelial dysfunction, angioparagraphgenesis, renal, and lung fibrosis (Duitama et al., 2020).

## 5 TRPM2 distribution and its role in various pathologies

TRPM2 is particularly gaining importance as it is permeable to both monovalent and divalent cations, as well as its activation/inhibition in response to various stimuli from both exogenous and endogenous sources, leading to changes in cellular responses that vary from picoseconds to several seconds. This is the only ion channel of the TRPM family associated with enzyme activity at NUDT9-H domain of the C-terminus sensitive to adenosine diphosphate ribose (ADPR) (Sumoza-Toledo and Penner, 2011; Perraud et al., 2001) thereby involving multiple functional roles like Ca^2+^ homeostasis (Hasan and Zhang, 2018), redox potential (Song et al., 2016), osmotic regulation, temperature sensitive gating (Vilar et al., 2020; Tan and McNaughton, 2018), production of inflammatory mediators (Yamamoto and Shimizu, 2016; Akyuva and Nazıroğlu, 2020), pain modulation (Di et al., 2012; Yüksel et al., 2017), cellular migration, cytoskeleton remodeling (Schwab et al., 2012), and immunity (Knowles et al., 2013; Syed Mortadza et al., 2015). Functional role of TRPM2 have been reported in many diseases such as Alzheimer’s and Parkinson’s disease, ischemic stroke, neuronal cell death, neurovascular functional injury, myocardial ischemia/reperfusion injury, vascular dysfunction, pancreatic β-cell death associated with pancreatitis, acute and chronic diseases, and liver toxicity (Malko and Jiang, 2020). TRPM2 channel is widely expressed in immune cells, microglia, neutrophils, T-lymphocytes. It also acts as a sensor for ROS and plays a key role in the inflammatory response in both normal and pathological states. Since TRPM2 has multifunctional role in oxidative stress and inflammation it would be worth exploring its mechanisms in attenuating pain-related diseases.

### 5.1 TRPM2 in cerebral ischemic stroke

TRPM2 is widely expressed in neurons and its role in ischemic stroke was evaluated in several *in vitro* and *in vivo* studies. TRPM2 have shown to increase the cell survival and inhibit apoptosis by various mechanisms (Ali et al., 2023; Shi et al., 2021). The following few studies represented the role of TRPM2 in ischemic stroke models.TRPM2 in primary cortical cultures of rat subjected to H_2_O_2_ induces apoptosis, and this is significantly reversed by using TRPM2 siRNA by inhibiting Ca^2+^ influx and cell death (Kaneko et al., 2006). The role of TRPM2 in oxidative stress induced by hypoxia has been studied in different animal models. TRPM2 KO mice, when subjected to transient ischemia by carotid artery occlusion, showed a 40% reduction in infarct volume. Some of the molecular pathways that contribute to cell survival are activation of protein kinase B (Akt) pathway and inhibition of glycogen synthase kinase-3β (GSK3β) pathway in TRPM2 KO mice (Alim et al., 2013). Genetic deletion of TRPM2 shows neuroprotective effects and improved sensory and motor outcomes in neonatal mice when compared to the wild type. TRPM2 KO mice showed an increased pro-survival signaling pathway and produced neuroprotective effects through Akt/GSK3β pathway (Huang et al., 2017). The use of TRPM2 inhibitors such as flufenamic acid (Nazıroğlu et al., 2007), clotrimazole (Hill et al., 2004), aminoethoxy diphenyl borate (APB) (Togashi et al., 2008), *N*-(*p*-amylcinnamoyl)anthranilic acid (ACA) (Kraft et al., 2006), and TRPM2 shRNA significantly reduced neuronal cell death following oxygen-glucose deprivation in males (Jia et al., 2011). Pharmacological inhibition of TRPM2 inhibits the adhesion of neutrophils in ischemic conditions (Gelderblom et al., 2014). There is a strong correlation between TRPM2 oxidative stress, inflammation, and ischemia followed by reperfusion injury (Huang et al., 2024).

### 5.2 TRPM2 and microglia

Apart from neuronal cells, TRPM2 is widely expressed in non-neuronal cells such as microglia and astrocytes (Turlova et al., 2018). Hypoxia-induced generation of H_2_O_2_ activated microglia increased TRPM2 mediated Ca^2+^ conductance in middle cerebral artery occlusion model injury (Patel et al., 2013). H_2_O_2_-induced oxidative stress revealed upregulation of TRPM2-like conductance and was reversed by flufenamic acid (Fonfria et al., 2006) in rat microglia. An increase in TRPM2 activity is associated with the generation of ROS, leading to the activation of Poly adenosine-diphosphate ribose polymerase 1 and TRPM2 activity were suppressed by inhibiting PKC and Nicotinamide Adenine Dinucleotide Phosphate (NADPH) oxidase (NOX) and downstream Mitogen-Activated Protein Kinase (MAPK)/extracellular signal-regulated kinase (MEK/ERK) pathway (Alawieyah Syed Mortadza et al., 2018). Release of cytokine interleukin-1 beta (IL-1β) from microglia is also mediated through TRPM2-dependent activation of Nucleotide-binding Oligomerization Domain-like receptor pyrin domain 3 inflammasome (Aminzadeh et al., 2018). Selenium, an important essential element through the GSH (Glutathione) peroxidase pathway, prevents interferon-gamma (IFN*γ*) induced activation of TRPM2 channel-mediated apoptosis in microglia (Akyuva et al., 2021). TRPM2 (knock out) KO in microglia suppressed the kainic acid-induced glial activation, cytokine production, and hippocampus paroxysmal discharges, affirming the role of TRPM2 via AMPK and mTOR pathway in epileptogenesis (Yıldızhan and Nazıroğlu, 2020). When stimulated with interferon-γ, microglia in the brain of C57bl/6 mice displayed excessive currents of TRPM2 (Akyuva et al., 2021). Macrophage exposure to LPS triggers the activation of TRPM2, thereby promoting NO production by regulating MAPK and JNK signaling pathways (Miyake et al., 2014).

### 5.3 TRPM2 in renal injury and fibrosis

Reactive oxygen species formed by renal ischemia-induced hypoxia and reperfusion activate TRPM2 ion channels. TRPM2-KO mice have shown resistance to renal injury (Khanahmad et al., 2022). Administration of TRPM2 antagonist, 8-bromo cADPR in Wistar rats shown to inhibit renal ischemia-reperfusion injury via caspase 3 inhibition (Eraslan et al., 2019). Cisplatin induced nephrotoxicity is also reduced by the activation of TRPM2 mediated autophagy (Yu et al., 2023). TRPM2-KO mice markedly improved renal dysfunction and its ablation remarkedly suppressed TGFβ mediated JNK activation renal fibrosis (Wang Y. et al., 2019). Thus, TRPM2 highlights its role in renal ischemic injury and fibrosis which is elevated during renal ischemic/reperfusion injury (Gao et al., 2014). Ischemia reperfusion-activated NOX and RAC1 were activated in WT mice but not in TRPM2-KO mice (Gao et al., 2014). Curcumin also reduces the albumin-evoked Ca^2+^-induced oxidative stress through the TRPM2 channel in renal tubules (Nazıroğlu et al., 2019).

### 5.4 TRPM2-mediated apoptosis

ROS-dependent neuronal inflammation and death involve multiple mechanisms, including pyro-apoptosis, autophagy, and apoptosis. ROS-mediated TRPM2 inhibits autophagy through downregulation of AMPK-induced Mammalian rapamycin target protein (mTOR), leading to ischemic/reperfusion-induced neuronal cell death (Hu et al., 2021). TRPM2 channels regulate cation permeability across the cell membrane that includes Zn^2+^ and the accumulation of the Zn^2+^ intracellularly induces ROS production, triggering lysosomal dysfunction and an increase in neuronal cell death (Ye et al., 2014). In addition, TRPM2 activates Calmodulin-dependent kinase II -mediated phosphorylation of Beclin1 that inhibits autophagy and induces neuronal death (Wang et al., 2016). TRPM2 mediated Ca^2+^ induced neuronal death was modulated by duloxetine preventing from apoptosis in hippocampus and dorsal root ganglion (DRG) of rats (Demirdaş et al., 2017).

### 5.5 TRPM2 in autoimmune disorders

The role of TRPM2 in inflammation has been extensively evaluated for Ca^2+^ mediated oxidative stress (ROS) that, in turn, activates innate immunity, thus delineating its role in autoimmune disorders such as rheumatoid arthritis, type 1 diabetes, multiple sclerosis, inflammatory bowel disease through multiple mechanisms. In an animal model of multiple sclerosis, TRPM2-KO or pharmacological inhibition of TRPM2 inhibits the progression of the disease. Moreover, decreased neutrophil infiltration in the central nervous system was observed in KO mice than in WT (Tsutsui et al., 2018). A decrease in neuronal antioxidant glutathione triggers the increased current of the TRPM2 channel (Belrose et al., 2012). Additional mechanisms such as ROS-ADPR- Poly adenosine diphosphate ribose polymerase mediated TRPM2 current in microglia thereby increase intracellular Ca^2+^ and thereby induce apoptosis of hippocampal pyramidal neurons in Alzheimer’s disease (Övey and Naziroğlu, 2015). TRPM2 also plays a key role in Amyloid beta (Aβ)/ROS-induced microglial-mediated neuroinflammation and neuronal death (Jiang et al., 2018). Glutathione depletion linked to oxidative stress induces apoptosis mediated through TRPM2 channels in microglial cells with Alzheimer’s disease model.

### 5.6 TRPM2 in hepatotoxicity

The liver is known as an important metabolic organ that regulates various functions. Acetaminophen is the commonly prescribed drug for pyrexia and is known to cause unwanted effects such as hepatotoxicity. ROS is the key mediator of acetaminophen-induced toxicity. Acetaminophen induced hepatocyte death by increasing Ca^2+^ influx in cultured rat and mouse hepatocytes which were blocked by ACA, clotrimazole or TRPM2-siRNA, and TRPM2-KO (Wang et al., 2016; Kheradpezhouh et al., 2014). TRPM2-mediated Ca^2+^ signalling activates Ca^2+^/CaMKII to inhibit autophagy (Ni et al., 2012). Also, acetaminophen-induced liver injury in WT mice was mitigated by treatment with CaMKII inhibitor KN-93 (Wang et al., 2016). In the hepatic ischemia-reperfusion injury model, TRPM2-mediated Ca^2+^ influx causes mitochondrial lipid peroxidation due to increasing arachidonate 12-lipoxygenase (Zhong et al., 2023). In addition, pretreatment with antioxidants such as N-acetyl cysteine and thymoquinone inhibits the Ca^2+^ entry by reducing the TRPM2 gene expression in the hepatocyte model of ischemia-reperfusion injury (Atilgan et al., 2022; Caglar et al., 2024).

## 6 TRPM2 expression and function in neuronal and non-neuronal pain

TRPM2 ion channels play an important role in generating, transmitting, and transforming nerve signals. Calcium ions play an important role in interneuronal communication, triggering an action potential and the release of certain neurotransmitters and mediators (Malko et al., 2019; Wang H. et al., 2019). Primary afferent neurons such as DRG and trigeminal ganglia also express TRPM2 channels (Vilar et al., 2020). The C-fibers of afferent neurons are mainly responsible for pain perception leading to depolarization. Substance P and calcitonin gene-related peptide (CGRP) of the peptidergic neurons, upon sustained stimulation, exacerbate tissue inflammation by infiltrating immune cells. TRPM2 is known to express abundantly in both peptidergic and non-peptidergic C-fibres (Matsumoto et al., 2016). TRPM2 is known to play a crucial role in afferent ganglia, where it responds to oxidative stress, particularly that caused by inflammatory injuries. This channel contributes to the sensory signaling processes by detecting and reacting to oxidative damage, which can occur during inflammation. The H_2_O_2_-induced inward currents in a whole cell patch clamp experiment with DRG neurons H_2_O_2_-induced inward currents were reversibly abolished by TRPM2 inhibitors 2-APB and ACA (Nazıroğlu et al., 2011; Naziroğlu et al., 2013). In addition, H_2_O_2_-mediated TRPM2 currents were inhibited by NADPH oxidase inhibitors such as apocynin and N-acetyl cysteine, which regulate the H_2_O_2_-ADPR-TRPM2 axis (Nazıroğlu, 2017). In cultured trigeminal ganglia upon H_2_O_2_ exposure, there is a significant increase in cytokine and chemokine levels which were prevented by treatment with TRPM2 inhibitors 2-APB (Chung et al., 2015). Therefore, TRPM2 plays an important role in the mechanisms of neuronal excitability and in the proinflammatory conditions under oxidative stress.

TRPM2 is widely expressed in immune cells. LPS stimulation of immune cells increased the expression of TRPM2, contributing to the production of cytokines (Wehrhahn et al., 2010). Also, neutrophil infiltration is linked with TRPM2 deficiency in carrageenan-induced inflammation (Haraguchi et al., 2012). IL- 1β secretion is also increased in macrophages by ROS-mediated TRPM2 activation (Zhong et al., 2013). The factors that regulate the calcium gating through TRPM2 include adenosine dinucleotide, cytokines, ROS, and intracellular calcium ions (Wang H. et al., 2019). Increased expression of TRPM2 in macrophages is associated with acceleration of inflammatory signals that regulate the pathophysiology of pain (Jang et al., 2018). Neuronal cell response to LPS is dependent on Ca^2+^, but the mechanisms involved are poorly elucidated. LPS is known to activate TLR-4, which initiates to activate of the downstream pathways of inflammation, such as phosphorylation of MAPKs, NF-kB translocation to the nucleus, and upregulation of inflammatory genes for cytokines such as TNFα, IL-1β, and IL-6 as shown in Figure 1. There is also evidence that LPS-mediated TLR-4 activates PLC, thereby mobilizing intracellular Ca^2+^. Thus, the role of Ca^2+^ and the production of cytokines are interlinked. Then, it comes to the question that controlling Ca^2+^ entry into the cell decreases inflammation. To understand this, the cytokine production generates ROS from mitochondria via modulating NADPH redox system is to be studied. The cytokine production generates ROS by activating NADPH oxidase, disrupting mitochondrial function, and activating MAPK pathways, including inducible nitric acid synthase and activating inflammasomes, contributing to the inflammatory response and cellular signaling (Bordt and Polster, 2014). The generation of free radicals takes place by intracellular production of H_2_O_2_ (Checa and Aran, 2020). Cellular activity sensors such as ion channels, particularly TRPM2, regulate the cation influx, including Ca^2+^, which further increases the inflammation process. Since regulation of inflammation is Ca^2+^ dependent which is also regulated by TRPM2 ion channel. Given the importance of TRPM2 ion channel in current scenario, modulating the function of TRPM2 is warranted. TRPM2 modulation with antioxidant compounds will alleviate the inflammation, thereby reducing nociception. Table 2 provides a summary of some research studies that use oxidative stress and inflammation as a model to modulate neuronal pain via TRPM2.

**TABLE 2:** Evidence of TRPM2 mediated neuropathic pain in response to inflammation and oxidative stress.

Cell Type	Inducer/Animal Model	Associated Pathology	Key observations	Ref.
Dorsal Root Ganglia (DRG) of rats	Spinal cord injury and Sciatic nerve injury	Neuronal death and apoptosis	*Hypericum perforatum* attenuates oxidative stress and apoptosis induced by SCI and SNI and, thereby, reducing, Ca^2+^ uptake through TRPM2	Özdemir et al. (2016)
TRPM2-KO mice	LPS	bladder inflammation	Lacking TRPM2 attenuates LPS-induced inflammation and hypersensitivity	Kamei et al. (2021)
Hippocampal neuron/TRPM2-KO mice	Morphine	Inflammation and apoptosis	Morphine Induces Apoptosis, Inflammation, and Mitochondrial Oxidative Stress via Activation of TRPM2 Channel and Nitric Oxide Signaling Pathways	Osmanlıoğlu et al. (2020)
C57/BL6 mice	LPS	Inflammation-induced cognitive impairment	IL-1β or TRPM2 level knockdown helped counter the cognitive impairment caused by significant inflammation	Yang et al. (2024)
Sciatic nerve of rats	streptozotocin	Diabetic neuropathic pain	Hesperidin treatment attenuated diabetes-induced neuropathic pain by reducing TRPM2 channel activation	Bayir et al. (2023)
Microglia from WT and TRPM2KO	LPS/IFNγ	Inflammation	TRPM2-mediated Ca^2+^ signaling is necessary to activate p38 MAPK and JNK signaling and results in increased NO production in microglia	Miyake et al. (2014)
TRPM2-KO	Capsaicin/H_2_O_2_/acetic acid	Inflammation	Acetic acid-induced writhing was significantly attenuated in TRPM2-KO	So et al. (2015)
Cultured DRG	Rotenone	Neuropathy	ADPR and rotenone-induced inward electrical currents were reversibly abolished by the TRPM2 inhibitors, APB, and ACA	Nazıroğlu et al. (2011)
Hippocampal and DRG of Wistar rats	Middle cerebral artery occlusion/reperfusion	Apoptosis	Dexamethasone shows remarkable neuroprotective impairment effects in the hippocampus and DRG of ischemia-induced rats by reducing Ca^2+^ via TRPM2	Akpınar et al. (2016)
Cultured rat Trigeminal Ganglia (TG)	H_2_O_2_	Inflammation	TRPM2 role in H_2_O_2_-induced expression of inflammatory cytokines was studied	Chung et al. (2015)
Rat DRG	ADP-ribose and H_2_O_2_	Neuropathic pain	NADPH oxidase-dependent activation of TRPM2	Nazıroğlu and Braidy (2017)
Human Monocytes	LPS	Cytokine production	TRPM2 is required for LPS-induced cytokine production	Wehrhahn et al. (2010)
Mice macrophages	Carrageenan	Inflammatory and neuropathic pain	TRPM2-KO mice develop less severe allodynia	Haraguchi et al. (2012)

The role of TRPM2 in acute inflammatory mechanisms is acknowledged not only in neuronal cells but also in nonneuronal cells. TRPM2 channels are widely expressed in various cell types, including microglia (Mortadza et al., 2017; Kraft et al., 2004), macrophages (Zou et al., 2013), neurons (Kaneko et al., 2006; Alim et al., 2013), endothelial cells (Hecquet and Malik, 2009; Mittal et al., 2017; Yang et al., 2006), cardiomyocytes (Yang et al., 2006), dendritic cells (Sumoza-Toledo et al., 2011), and pancreatic beta cells (Ishii et al., 2006; Togashi et al., 2006). This indicates that TRPM2 plays multiple functional roles in many physiological and pathological processes. TRPM2 has been shown to regulate intracellular calcium levels through lysosomal, endoplasmic reticulum, and mitochondrial compartments through various pathways that mediate through both exogenous and endogenous mediators (Zhang et al., 2018).

TRPM2 is known to be regulated by other receptor mechanisms in inflammation and pain, such as the N-methyl-D-aspartate (NMDA) receptor (NMDAR), which activates to promote TRPM2-mediated Ca^2+^ influx via ERK1/2-dependent PARP-1 recruitment of microglia-dependent neuroinflammation (Raghunatha et al., 2020). TRPM2 channels can also be activated through intracellular signaling cascades initiated by pruritogen receptors and, thereby, neuronal activation. TRPM2 via Protein Kinase C gamma (PKC-*γ*), a neuro-specific PKC phosphorylates serine/threonine residues, activates and increases the expression of NMDAR, thereby increasing the excitotoxicity of NMDARS, and the interaction between them is thought to be mediated by oxidative stress (Sun and Alkon, 2014). Antioxidants such as Selenium reduced the fibromyalgia induced increase in TRPM2 and TRPV1 currents, pain intensity, ROS, and intracellular free Ca^2+^ in sciatic and DRG neurons (Yüksel et al., 2017).

## 7 Toll-like receptors

Toll-like receptors (TLRs) are pattern recognition receptors embedded in the phospholipid membrane as well as endosomes and lysosomes (Sahoo, 2020). In humans, there are ten TLRs (TLR1-TLR10), but overall, there are 12 TLRs. Different TLRs recognize and activate different pathogen-associated molecular patterns (PAMPs) (Kawai and Akira, 2007). TLR-1 and TLR-2 link together and recognize bacteria lipoproteins which activate immune responses. TLR-2 not only forms a heterodimer with TLR-1 but also with TLR-6 to recognize microbial patterns. TLR-3 is found embedded in endosomes, senses viral infections, and activates the production of type-I interferons. TLR-4 is activated by bacterial LPS, which is the main cell wall component of Gram-negative bacteria (Kuzmich et al., 2017). This activation can contribute to inflammation and pain pathways, leading to the release of pro-inflammatory cytokines and other mediators that contribute to the development or exacerbation of neuropathic pain. Some Gram-negative bacteria associated with this process include *Escherichia coli* (*E. coli*), *Pseudomonas aeruginosa*, *Klebsiella pneumoniae*, *Salmonella* spp., *Neisseria* meningitidis, *Haemophilus* influenzae, *Helicobacter pylori* (Deng and Chiu, 2021; Staurengo-Ferrari et al., 2022). TLR4 can also be activated by a variety of non-LPS ligands. These ligands include endogenous danger-associated molecular patterns (DAMPs) and exogenous pathogen-associated molecular patterns (PAMPs) other than LPS (Facchini, 2018; Alexzander Asea et al., 2002; Erridge, 2010). TLR-7, TLR-8, and TLR-9 are located in endosomes and lysosomes, while TLR-5, found on the cell surface, recognizes bacterial flagellins involved in motility, leading to the activation of inflammatory cytokines (Tegtmeyer et al., 2020). They are mainly expressed in various immune cells and initiate inflammatory responses. TLR-10, although found on both the cell surface and endosomes, the actual functions are not well-defined (Noreen and Arshad, 2015).

However, Toll-like receptors are a group of receptors that recognize and initiate innate and adaptive immune responses. Like other receptors, they span cellular transmembrane with an external domain of leucine repeats and cytosolic Toll interleukin-1 receptor (TIR) domains to activate downstream pathways. The external domain tends to recognize endotoxins, heat shock proteins, cellular damage products, and High mobility group box 1 (HMGB-1) proteins (Bettoni et al., 2008). The receptors are widely distributed not only on antigen-presenting cells but also on myocytes, adipocytes, thyroid cells, mesangial cells, and fibroblasts. The receptors are also expressed in sensory neurons, microglial cells, and astrocytes (Vaure and Liu, 2014). TLR-4 receptor undergoes dimerization with myeloid differentiation protein-2 upon ligand binding to microdomain lipid rafts and initiates TIR adoption molecules, thereby initiating a signaling cascade.

TLR-4, through the TIR domain-containing adapter-inducing Inhibitor of the kappa B (IkB) pathway, induces NF-kB and expression of proinflammatory cytokines such as IL-1, IL-6, and TNF-α. Activation and inhibition of these pathways create a balance between the production of these cytokines and Interferon Type-1 (IFN-1) (Roy et al., 2016; Kawasaki and Kawai, 2014).

## 8 Role of TLR-4 in the regulation of neuronal pain and inflammation

TLR4 plays an important role in adaptive immune response, induction, and maintenance of acute and chronic pain states. Recognition of pathogens and products from damaged neurons by microglia leads to activation of TLR-4 and increased expression of proinflammatory mediators. Sustained activation or dysregulation of TLR-4 contributes to inflammatory and autoimmune diseases such as Crohn’s Disease, atherosclerosis, rheumatoid arthritis, type 1 diabetes, and other neurodegenerative diseases (Takeda et al., 2003; Booth et al., 2011; Hosseini et al., 2015).

Microglial activation of TLR-4 after spinal cord injury is pivotal for the induction of pain by modulating proinflammatory cascade and expression of TNFα, IFN-γ, IL-6, IL-1β, and NF-kB (Kuang et al., 2012; Stokes et al., 2013). In rat animal model of chronic constriction injury TLR-4 antagonists and siRNA-mediated suppression of spinal cord TLR-4 signaling prevent the activation of the NF-kB pathway and production of proinflammatory cytokines, thereby attenuating allodynia and thermal hyperalgesia, emphasizing its preventive role in neuropathic pain (Eidson and Murphy, 2013; Wu et al., 2010). TLR-4 also mediates the conversion of acute to chronic pain. Intrathecal administration of TLR-4 antagonists reversed the chronic constriction injury-induced thermal hyperalgesia and mechanical allodynia in wild-type mice, whereas in knockout and point mutant mice, attenuation of thermal hypersensitivity along with spinal microglial activation and lower proinflammatory cytokines suggesting its role in the maintenance of chronic pain (Hutchinson et al., 2008; Christianson et al., 2011; Nicotra et al., 2012; Woller et al., 2017). Administration of TLR-4 antisense oligonucleotide prevents thermal hypersensitivity with reduced expression of mRNA for microglial markers and spinal proinflammatory cytokines (Tanga et al., 2005).

Hydrogen peroxide, a ROS, oxidatively modifies TLR-4 proteins, and it mimics LPS binding (Powers et al., 2006). It activates the release of damaged-associated molecular proteins (DAMPs) from dying cells, which trigger TLR-4 proteins (Shim et al., 2017). H_2_O_2_ also modulates the activity of kinases and transcription factors such as NF-κB and MAPKs, which are critical for TLR-4 signaling, which can amplify inflammatory responses (Burgueno et al., 2021). When H_2_O_2_ production is increased, it acts as a secondary signal to aid TLR-4-mediated defenses guaranteeing an immune response (Zhou et al., 2013). The interaction between ROS and TLR-4 can intensify inflammation indicating tissue damage and disease development (Gunawardena et al., 2019).

## 9 Relationship between TRPM2 and TLR-4 in neuropathic pain

The generation of neuropathic pain involves complex pathways within the nervous system, both peripheral and central. The process is initiated by damage or dysfunction in the somatosensory nervous system, which leads to abnormal pain signaling. Neuropathic pain often involves nerve injury or chronic inflammation, leading to increased oxidative stress and inflammatory mediators such as proinflammatory cytokines, chemokine, and ROS, which can activate TRPM2 channels. Understanding the mechanisms by which TRPM2 contributes to neuropathic pain can provide insights into potential therapeutic targets for managing chronic pain conditions.

On the other hand, TLR-4 is a key player in the immune system, primarily recognized for its role in detecting pathogens and initiating inflammatory responses. However, TLR-4 plays a critical role in the pathogenesis of neuropathic pain by driving neuroinflammation, central sensitization, and the maintenance of a chronic pain state. Targeting TLR-4 or its downstream signaling pathways is a potential therapeutic strategy for managing neuropathic pain.

TLR-4 is expressed on the cell membrane and senses pathogen-associated molecular patterns such as LPS, peptidoglycans, chitin, and glucans (Brown et al., 2011). Upon recognition and activation, TLR-4 recruits adaptor molecules such as MyD88 and intracellular Toll-interleukin (IL)-1 receptor domains and promotes downstream pathways leading to the secretion of proinflammatory mediators, including cytokines (Blasius and Beutler, 2010; Santoni et al., 2015). These proinflammatory responses by the immunocompetent cells in the brain, such as microglia, contribute to neuroinflammation leading to pain. In chronic states such as neuropathic pain, microglial activation results in deleterious consequences (Block et al., 2007). Microglia express a wide variety of ion channels, including TRP channels, that are necessary for cytokine production, proliferation, and migration of microglia (Echeverry et al., 2016). Studies indicate that H_2_O_2_-induced TRPM2 activation mediates Ca^2+^ influx, which modulates physiological and pathological cellular functions (Shirakawa and Kaneko, 2018). TRPM2 is expressed in both neurons and glia, and oxidative stress-induced TRPM2 activation is implicated in neuronal diseases (So et al., 2015). Miyake et al. (2014) demonstrated that LPS and IFN-γ can stimulate TRPM2 mediated Ca^2+^ in microglia. They also showed that activation of TRPM2 results in increased NO production. Interaction between nociceptive neurons and glial cells plays an important role in neuropathic pain (Ren and Dubner, 2010).

TRPM2, a calcium-permeable cation channel present in sensory neurons, is capable of recognizing mechanical, chemical, and thermal stimuli (Santoni et al., 2015). TRPM2 activation leads to increased calcium levels, which also results in a pain-signaling response in the sensory neurons. TLR-4 has been linked to neuroinflammation (Table 3) and the sensitization of nociceptors (Malko et al., 2019). When TLR-4 interacts with ROS, TLR-4 proteins can intensify inflammation and hypersensitivity to pain through the opening of TRPM2 channels. Thus, the pathways of both TRPM2 and TLR4 converge on neuroinflammation (Yamamoto and Shimizu, 2016). Neuropathic Pain is a chronic pain caused by a disease of the somatosensory nervous system (Finnerup et al., 2021). TRPM2 contributes to the release of pro-inflammatory cytokines (Haraguchi et al., 2012), and the production of cytokines, in turn, activates TLR-4, which plays an important role in neuropathic pain and induces inflammatory and immune responses (Kuzmich et al., 2017).

**TABLE 3:** Pain signaling pathways through TLR4 and TRP channels.

Pathological condition/disorders	Experimental paradigm	Treatment	Mechanism/pathways	Principal Effects/Remarks	Ref.
Neuropathic pain	Thermal and Mechanical Hypersensitivities in a Rat Model of Paclitaxel-Induced Peripheral Neuropathic Pain	Electro-acupuncture (EA)	NF-kappaB inhibitor alpha (NF-κB) and Mitogen-activated protein (MAP) kinase pathways	EA suppresses TLR4 and its downstream signaling molecule MyD88 overexpression in DRGs of paclitaxel-treated rats	Li et al. (2019)
Chronic Neuropathic Pain	The therapeutic effect of β-sitosterol on chronic neuropathic pain by performing behavioral tests on Sciatica models (chronic constriction injury on SD rats)	β-Sitosterol	β-sitosterol can affect microglial polarization by inhibiting the TLR4/NF-κB signaling pathway	β-Sitosterol Reduces Pain Sensitivity in the Right Hind Limb of Sprague‒Dawley Rats	Zheng et al. (2023)
Neuropathic pain	Therapeutic effect of ferulic acid on the chronic constriction injury induced pain rat model via the von Frey test and acetone experiment	Ferulic acid	The levels of IBA‐1, IL‐1β, iNOS, TLR4, Myd88, p‐NF‐κB, and p‐p38MAPK increased signaling pathway	Ferulic acid can promote injured sciatic nerve repair by reducing neuronal cell apoptosis and inflammatory infiltration though the TLR4/NF‐κB pathway	Zhang et al. (2023)
Migraine and Functional Gastrointestinal Disorders	Using a murine model of light aversion produced by compound 48/80. TLR4 in migraine-like behavior and neuronal activation	Compound 48/80	TLR4 utilizes both MyD88 and TRIF adapter protein pathways for downstream signaling	TLR4 signaling in migraine is the report that naloxone was reported to be effective in treating acute migraine attacks	Ramachandran et al. (2019)
Cancer-induced Neuropathic Pain	Cancer-induced neuropathy model and determined the thresholds of cold allodynia and thermal and mechanical hyperalgesia	Ruthenium Red	TRPV1 over TRPV4 antagonism is attributed to its multiple mechanisms of action and different pathways and target receptors other than the TRP channels	The increased expression of TLR4 and ERK1/2 reveals immune response and tumor progression, respectively, and their ultimate decrease is an indicator of nerve damage	Maqboul and Elsadek (2018)
Acute Inflammatory Visceral Pain (AIVP)	AIVP was used to examine the antinociceptive efficiency of DEX and assess its effects on the activation of the ERK and TLR4 signaling pathway and release of CGRP during visceral hypersensitivity	Dexmedetomidine (DEX)	TLR4 receptor and its downstream NF-κB and IRF3 were upregulated, and the phosphorylation of P65 and IRF3 also increased upon acetic acid treatment	Antinociceptive effects of DEX might be partially mediated via suppression of the inflammatory responses associated with AIVP	Liu et al. (2018)
Chronic Sciatica	The therapeutic effect of α-asarone on CCI of the sciatic nerve via behavioral tests, pathology, and immunohistochemistry	α-Asarone	α-Asarone decreased the mRNA levels of IL1β, TNF-α, TRPV1-4, TRPA1, and TRPM8	α-Asarone relieves chronic sciatica by decreasing the levels of inflammatory factors, inhibiting peripheral sensitization, and favoring the repair of damaged nerves	Zhang et al. (2023)
Multiple Sclerosis	Spinal cord alterations induced by this novel SD rat model of MOG-induced EAE, optimized to avoid motor impairments/disabilities	myelin-oligo dendrocyte-glycoprotein (MOG)	Ligation of TLR4 induces NF-kB activation that primes the NLRP3 inflammasome and can ultimately lead to the production of the proinflammatory cytokine IL-1β t	Doses of 8 and 16 μg MOG could produce long-lasting mechanical allodynia in the absence of motor impairments/disabilities in male SD rats	Kwilasz et al. (2022)
Rheumatoid Arthritis)	Molecular mechanisms of Baihu-Guizhi decoction (BHGZD), against active RA were validated by a series of experiments based on the adjuvant-induced arthritis-modified rat model	(BHGZD)	BHGZD suppress TLR4/PI3K/AKT/NFκB signaling-related protein activation, and subsequently inhibit NLRP3 inflammasome-induced pyroptosis	BHGZD effectively improved disease severity of active RA rats, elevating pain thresholds, relieving joint inflammation and bone erosion via inhibiting TLR4/PI3K/AKT/NFκB signaling to suppress the activation of the NLRP3 inflammasome	Li et al. (2022)
Interstitial cystitis/bladder pain syndrome (IC/BPS)	IC rat model by intraperitoneal injection of cyclophosphamide. MSC-EVs were isolated from the culture supernatants of human umbilical cord-derived MSCs	Mesenchymal stem cell-derived extracellular vesicles (MSC-EVs)	the expression levels of NLRP3, Caspase-1, IL‐1β and IL‐18 in the SDH were reduced	Injection of MSC-EVs can alleviate neuroinflammation and mechanical allodynia in IC rats by inhibiting NLRP3 inflammasome activation	Zhang et al. (2022)
Post-epidural fibrosis	murine laminectomy model to observe the effect of metformin on epidural scars	Metformin	Metformin inhibited the hyper-proliferation of epidural scars after laminectomy via the reduction in fibronectin and collagen deposition by inhibiting the HMGB1/TLR4 and TGF‐β1/Smad3 signaling pathway	metformin may be a potential therapeutic option to mitigate epidural fibrosis after laminectomy	Song et al. (2021)

TRPM2 channel is a newly cited player for neuroinflammation mediated in response to LPS, a classical target for TLR-4 activation where the interaction between TLR-4 and TRPM2 in neuronal cells was least explored. TLR-4-mediated responses required Ca^2+^ influx through TRPM2 (Schappe et al., 2018; Tauseef et al., 2012), and the binding of LPS to TLR-4 activation increases the production of diacylglycerol (DAG) and the subsequent increase in Ca^2+^ concentration, resulting in MyD88-NF-kB activation (Mohammadi, 2019). TRPM2-mediated Ca^2+^ entry is implicated in inflammatory responses in neuronal and non-neuronal cells (Tauseef et al., 2012; Mehta et al., 2003). LPS-TLR-4 activation triggers NF-kB-mediated cytokine production resulting in TRP-mediated Ca^2+^ influx. Cytokine increases intracellular Ca^2+^ concentration, thereby inducing early ROS production preceded by TRPM2-mediated Ca^2+^ influx (Simon et al., 2017; López-Requena et al., 2017). Consequently, TRPM2 regulates total ROS production and Ca^2+^ influx. The existing studies suggest TRPM2 is not only involved in physiological nociceptive pain, but also in inflammatory and neuropathic pain (So et al., 2015).

The role of TLR-4-induced inflammation via calcium channels has been reported. Activation of TLR-4 through LPS-induced cytokine production results in an imbalance of basal calcium and store-operated calcium channels, particularly TRPM2, in microglia through Orai-1, a downstream protein of TLR4-MAPK (Birla et al., 2022). Various proinflammatory cytokines are produced during the immune response to an infection. The absence of TRPM2 channels improved neuroethology and pathological changes and decreased inflammatory cytokines and apoptosis proteins in TRPM2 knock-out mice in the LPS-induced sepsis model associated with encephalopathy infection (Zhu et al., 2019). In a study, where the LPS-induced production of IL-6, IL-8, and TNF-α in THP1 monocytic cells was significantly attenuated by using shRNA to reduce TRPM2 expression (Wehrhahn et al., 2010). Also, increased cell-free heme activates TLR4-mediated ER stress and ROS leading to spinal microglial activation and neuroinflammation, contributing to acute and chronic pain (Lei et al., 2021). Ketamine, an anesthetic agent, attenuated hypoxia-induced TRPM2-mediated Ca^2+^ influx, ROS, NMDAR, and ROS in neuronal cell (SH-SY5Y) death (Osmanlioğlu, 2022).

There is a reciprocal relationship between neuroinflammation and oxidative stress. These two processes are not independent but rather complex reciprocal interactions (Teixeira-Santos et al., 2020). NOX activity upregulates the expression of proinflammatory cytokines and proinflammatory cytokines increase ROS through NOX (Kallenborn-Gerhardt et al., 2013). Initiation of TLR4 downstream pathway requires NOX activity (Haslund‐Vinding et al., 2017) and TLR-4 receptors may be required for NOX expression (Lim et al., 2013), thus both are dependent on each other. These interdependent complex mechanisms that regulate neuropathic pain require Ca^2+^ ion, which is well controlled by the TRPM2 ion channel sensitive to cellular redox potential (Ogawa et al., 2016), and hence modulated TRPM2 can alleviate neuropathic pain.

NOX 2 and NOX 4 have been implicated in chronic pain mechanisms such as neuropathic pain (Grace et al., 2016). Inhibition of NOX and its isoforms 2 and 4 have shown to be beneficial in neuropathic pain models (Kallenborn-Gerhardt et al., 2013). Macrophages produce ROS during immune response, and it is well regulated by NOX. This enzyme activity can be regulated by Ca^2+,^ which is controlled by protein kinase C α and membrane potential (Bedard and Krause, 2007). However, TRPM2KO mice showed greater mortality than WT in LPS-induced inflammation (Di et al., 2012). In addition, TRPM2 deficiency reduces oxidized low-density lipoprotein uptake by macrophages, thereby minimizing macrophage infiltration and inflammatory response by modulation of NOX (Zong et al., 2022).

However, the molecular mechanism underlying the interaction between TRPM2 and TLR4s and the quantum of impact in acute and chronic neuropathic pain remains unclear. It would be worth focusing on TRPM2 interaction with TLR4 in the context of neuropathic pain. Mechanistic studies that focus on TLR4-TRPM2 interaction will open new pathway interactions for treating inflammation-associated diseases such as neuropathic pain.

## 10 Conclusion

This review summarizes the functional role and mechanisms of TRPM2 that mediate Ca^2+^ influx under oxidative stress and inflammation linked with TLR-4. The reported literature in this review demonstrates the role of the TRPM2 ion channel in various pathological states and, more importantly, neuropathic pain. Many research findings support the evidence that oxidative stress and LPS-induced TLR4-mediated inflammation exert their effects mediated by the TRPM2 channel in neuronal and non-neuronal tissues. The existing and growing scientific evidence of TRPM2 in various pain pathologies has made researchers and the pharmaceutical industry focus on the development of novel targets for diagnostic and therapeutic approaches for pain treatment. This review also demonstrates the role of various inhibitors/drugs that modulate neuropathic pain in various disease states. However, the role of TRPM2 ion channels and their association with Toll-like receptors in pain management needs to be studied in more detail for the development of effective strategies to treat neuropathic pain.

## Figures and Tables

**Table:** 

**ACA**	*N*-(*p*-amylcinnamomyl) Anthranilic acid
**ADPR**	Adenosine diphosphate ribose
**AIVP**	Acute inflammatory visceral pain
**AKT**	Protein kinase B
**AMPK**	Adenosine 5′-monophosphate (AMP)-activated protein kinase
**APB**	Aminoethoxy diphenyl borate
**Aβ**	Amyloid beta
**BHGZD**	Baihu-Guizhi decoction
**BPS**	Bladder pain syndrome
**cADPR**	cyclic ADP-ribose
**CGRP**	Calcitonin gene related peptide
**DAG**	Diacyl glycerol
**DAMPS**	Damage-associated molecular patterns
**DEX**	Dexmeditomidine
**DRG**	Dorsal root ganglia
**ER**	Endoplasmic reticulum
**ERK**	Extracellular signal-regulated kinase
**GSH**	Glutathione
**GSK3β**	Glycogen synthase kinase-3β
**H**_**2**_ **O**_**2**_	Hydrogen peroxide
**HMGB-1**	High mobility group box 1
**IFN-1**	Interferon Type 1
**IkB**	Inhibitor of kappa B
**IL-1β**	Interleukin-1 beta
**IL-6**	Interleukin-6
**IP3**	Inositol triphosphate
**JNK**	c-Jun N-terminal kinase
**KO**	Knock Out
**LPS**	Lipopolysaccharide
**MAPKs**	Mitogen-Activated Protein Kinase
**MOG**	Myeloid oligodendrocyte glycoprotein
**MSC**	Mesenchymal stem cell
**mTOR**	Mammalian rapamycin target protein
**Myd88**	Myeloid differentiation Primary Response
**NADPH**	Nicotinamide Adenine Dinucleotide phosphate
**NF-kB**	Nuclear factor kappa B
**NMDA**	N-methyl D-aspartate
**NOX**	NADPH oxidase
**NUDT9-H**	Nudix hydroxylase
**PAMPS**	Pathogen-associated molecular patterns
**PARP1**	Poly adenosine diphosphate ribose polymerase
**PI**	Phosphatidyl inositol
**PKC**	Protein Kinase C
**PLC**	Phospholipase C
**ROS**	Reactive Oxygen Species
**SCI**	Spinal Cord Injury
**SD**	Sprague Dawley
**TGFβ**	Tumor growth factor
**TIR**	Toll interleukin −1 receptor
**TLR**	Toll-like-receptor
**TNFα**	Tumor necrosis factor α
**TRAF6**	Tumor necrosis factor receptor (TNFR)-associated factor 6
**TRIF**	TIR (Toll/interleukin-1 receptor) domain-containing adaptor protein
**TRP**	Transient receptor potential
**TRPA**	Transient receptor potential Ankyrin
**TRPC**	Transient receptor potential Canonical
**TRPM**	Transient receptor potential Melastatin
**TRPV**	Transient receptor potential Vanilloid
**WT**	Wild Type
